# Validation of the VEINES-QOL quality of life instrument in venous leg ulcers: repeatability and validity study embedded in a randomised clinical trial

**DOI:** 10.1186/s12872-015-0080-7

**Published:** 2015-08-11

**Authors:** J. Martin Bland, Jo C. Dumville, Rebecca L. Ashby, Rhian Gabe, Nikki Stubbs, Una Adderley, Arthur R. Kang’ombe, Nicky A. Cullum

**Affiliations:** Deparment of Health Sciences, University of York, York, YO10 5DD UK; School of Nursing, Midwifery and Social Work, University of Manchester, Oxford Road, Manchester, M13 9PL UK; Leeds Community Healthcare NHS Trust St Mary’s Hospital, Greenhill Road, Armley, LS12 3QE UK; School of Health and Social Care, Baines Wing, University of Leeds, Leeds, LS2 9JT UK; Liverpool School of Tropical Medicine, Pembroke Place, Liverpool, L3 5QA UK

## Abstract

**Background:**

VEINES-QOL/Sym is a disease-specific quality of life instrument for use in venous diseases of the leg. Its relative scoring system precludes comparisons between studies. There were very few venous leg ulcer patients in the validation samples. We report a validation study for venous leg ulcers and develop a scoring system which enables comparison between studies.

**Methods:**

Four hundred fifty-one participants in the VenUS IV trial of the management of venous leg ulcers were asked to complete a VEINES-QOL questionnaire at recruitment, along with SF-12, pain, and other aspects of quality of life. VEINES-QOL was repeated after two weeks and after 4 months. Healing of ulcers was confirmed by blind assessment of digital photographs. Three scoring systems for VEINES-QOL were compared.

**Results:**

No floor or ceiling effects were observed for VEINES-QOL items, item-item correlations were weak to moderate, item-score correlations were moderate. Internal reliability was good. The VEINES-Sym subscale was confirmed by factor analysis. Test-retest reliability was satisfactory for the scale scores; individual items showed moderate to good agreement. Relationships with SF-12, pain, and the quality items confirmed construct validity. Participants whose ulcers had healed showed greater mean increase in scores than did those yet to heal, though they continued to report leg problems. An intrinsic scoring method appeared superior to the original relative method.

**Conclusions:**

VEINES-QOL was suitable for use in the study of venous leg ulcers. The intrinsic scoring method should be adopted, to facilitate comparisons between studies.

**Trial registration:**

VenUS IV is registered with the ISRCTN register, number ISRCTN49373072.

## Background

It has been suggested that quality of life is really the right primary outcome variable for a clinical trial (Jon Nicholl, personal communication). Generic quality of life measures, such as SF-36, are necessary to compare outcomes across different populations and interventions, particularly for cost-effectiveness studies, but they are often insensitive to specific clinical changes which treatments are designed to bring about [1, 2]. Disease-specific quality of life measures may be more sensitive for the detection and quantification of changes that are important to clinicians or patients. As a result, comparison studies are needed of the validity, reliability, and responsiveness of generic and disease-specific measures in the same population [2].

### Venous leg ulcers

Venous leg ulcers are chronic wounds that generally occur in the region of the leg between the knee and ankle, as a consequence of venous insufficiency [3]. The underlying venous insufficiency and associated venous hypertension are generally caused by venous valve dysfunction, deep vein occlusion or failure of the calf muscle pump [4, 5]. Venous leg ulceration typically presents as repeated cycles of ulceration, healing and recurrence, with ulcers typically taking weeks or months to heal [3, 6]. Once healed, 12-month recurrence rates have been estimated at between 18 and 28 % [7, 8]. These wounds are one of the most prevalent chronic wound types in the UK, with an estimated point prevalence of 0.16 % [9]. There is a progressive increase in venous leg ulceration with age and the annual UK prevalence in people of >65 years is estimated at 1.7 % [10].

Venous leg ulcers are distressing to patients, painful, prone to infection, malodorous and have a severe negative impact upon patients’ mobility [9]. These all affect sufferers’ quality of life, both directly through pain and indirectly through limitations on work capacity, social activity, self-care and personal hygiene, and through associated depression, anxiety, and social isolation [11].

### The VEINES-QOL instrument

The VEINES-QOL/Sym questionnaire is a disease-specific quality of life instrument for chronic venous disorders of the leg (CVDL). It had good psychometric properties when used with a mixed sample of people with venous leg diseases [12] and in people with deep vein thrombosis [13]. Although venous leg ulcer patients were included in the original validation study [12], they were only a small part (2 %) of the sample and results were not presented for them separately. We would like to know how well VEINES-QOL/Sym can measure disease-specific quality of life in patients with venous leg ulcers.

The VEINES-QOL questionnaire consists of 26 items. It includes questions about symptoms due to CVDL (ten items), limitations in daily activities due to CVDL (nine items) and psychological impact (five items), as well as questions asking about the amount of change in the respondent’s leg problem over a 1-year period (one item) and the time of day that the leg problem is most intense (one item). The questions and coding are given by Lamping et al. [12].

Of the 26 items in the questionnaire, 25 items are combined to create a summary score (VEINES-QOL). One item which asks about the time of day the leg problem is most intense, question 2, provides only descriptive information and is not scored. A subset of ten of these items, questions 1a to 1i and 7, is used to create a symptom score (VEINES-Sym). For both the VEINES-QOL and VEINES-Sym scores, high values indicate better outcomes.

The way the items are combined to form a score is unusual. The scores for individual questions have varying numbers of possible answers and hence varying maximum scores, so they cannot simply be summed. Each question is standardised using the mean and standard deviation of the sample being coded to give a z-score, these are averaged, and the result transformed to T-scores (mean = 50; standard deviation = 10) [12, 14]. This is described as the method used for SF-36 [12]. In fact, SF-36 uses means and standard deviations obtained from a large sample of the general population to obtain T-scores, not those from the sample being studied [15]. Hence a calculated SF-36 score gives a number which is relative to this large reference sample and so we can compare SF-36 scores between studies. VEINES-QOL scores can be compared only to other members of the same sample. Each set of VEINES-QOL scores will have a mean T-score = 50. If we measure a sample on two occasions, the overall mean score will be identical, even if between the two occasions all the ulcers heal.

Missing items in the questionnaire are handled by averaging the remaining items after standardisation, providing that half of the questions or more are present. If they are not, the whole score is set to missing.

We tried to obtain a copy of the original validation data [12]. We regret to report that Prof. Lamping has died and we were unable to do so.

### Standardising VEINES-QOL

There are several approaches we could take to calculating the summary scores for VEINES-QOL:

1. We could use the original relative method [12, 14].
2. We could use an external standard for means and standard deviations, as used for SF36. It would not be possible to get a “normal” sample for this disease-specific instrument, because all the questions are about “your leg problem”.
3. We could use an intrinsic standard. All the questions are scored 1, 2, 3, . . ., *k*, where *k* is the number of categories. We could recode each item score *i* to (*i* – 1)/(*k* – 1) to give each item a score between 0 and 1 and average over the questions to give a final score.

This study has been designed to investigate several aspects of validity of the VEINES-QOL questionnaire for venous leg ulcers, including dimensionality and factor structure, internal consistency, effect of scoring method, construct validity, repeatability or test-retest reliability, and responsiveness to ulcer healing.

## Methods

### Participants

VenUS IV was a multi-centre randomised trial comparing compression delivered by two layer hosiery and by the four layer bandage system in the treatment of venous leg ulcers. The details of the design and results are given elsewhere [16, 17]. In brief, 454 participants with venous leg ulcers were recruited, randomised and followed-up over the course of their treatment. Table 1 shows the characteristics of participants at baseline. Healing was confirmed using digital photographs, by observers blind to treatment. Health-related quality of life and resource use data were collected at baseline and then by postal questionnaire at 3, 6, 9, and 12 months. The median time to healing was estimated to be 98 days and after 1 year of follow-up the Kaplan Meier estimate of the proportion healed was 82.6 %. There was no evidence of a difference in healing between the hosiery and bandage treatments. After adjustment for baseline ulcer area, duration, and mobility, with shared centre frailty effects, the hazard ratio for healing (hosiery/bandage) was 0.99 (95 % CI 0.79–1.25, *p* = 0.96) [17].

**Table 1:** VenUS IV participant characteristics at baseline

Number recruited	454^a^
Male (n (%))	230 (50.7 %)
Age (years) (Mean (SD))	68.6 (14.5)
Body mass index (kg/m^2^) (Mean (SD))	31.0 (8.0)
Mobility	
Participant walks freely (n (%))	289 (63.8 %)
Participant walks with difficulty (n (%))	160 (35.3 %)
Participant is immobile (n (%))	4 (0.9 %)
SF-12 quality of life score (Mean (SD))^b^	
Physical component	38.4 (11.2)
Mental component	49.6 (11.3)
Diabetes	78 (17.2 %)
Ulcer area (cm^2^) (median (IQR))	3.9 (1.6, 8.7)
Ulcer duration (months)) (median (IQR))	4.0 (2.0, 11.0)
Time since first ulcer (months) (median (IQR))	36.0 (4.0, 120.0)
Total ulcers on reference leg (n) (median (IQR))	1.0 (1.0, 2.0)
Reference leg	
Left (n (%))	256 (56.4 %)
Right (n (%))	198 (43.6 %)
Ankle mobility of reference leg (n (%))	
Participant has full range of motion	313 (68.9 %)
Reduced range of ankle motion	132 (29.9 %)
Participant’s ankle is fixed	9 (2.0 %)
Ankle brachial pressure index of reference leg (Mean (SD))	1.1 (0.1)

Key: IQR = Interquartile range, SD = Standard deviation

^a^For some variables a small number of observations are missing

^b^in general population, we expect mean = 50, SD = 10

We added the VEINES-QOL questionnaire to the baseline questionnaire battery. For this validation sub-study, we gave two additional postal questionnaires including only VEINES-QOL, after 2 weeks and at 4 months. In each case, we used the original wording [12].

### Validation methods

Several aspects of validation were considered. We asked whether all scale items were measuring aspects of the same underlying thing by checking that all were correlated with each other and with the overall composite scale. Any items which were unrelated to any others could possibly be omitted from the scale and any which were very closely related to others might be superfluous. We asked whether the scale was a single unified whole, or whether it might contain identifiable subscales which might represent different components of quality of life. We did this by factor analysis. We measured how well the items came together to form a scale using Cronbach’s alpha coefficient [18]. We asked whether it was related to those things to which we might expect quality of life to be related, convergent validity, and whether it was unrelated to things we should not expect to be related to quality of life, divergent validity. We asked how repeatable the score was, as a measurement which does not give consistent results on repeated measuring cannot be valid. We asked how responsive the score was, whether it changed when the respondent’s disease state changed.

### Individual items

Three VEINES-QOL questions, Q3, Q6, and Q7, have their scoring reversed before analysis. Question 4a of VEINES-QOL is about physical limitations for daily activities at work. One option is “I do not work”. We have followed the VEINES-QOL coding manual in setting question 4a to missing if this option is selected.

For each item, we estimated the percentage response to each possible answer, the mean and standard deviation of the numerical score for each question, and drew a histogram of the distribution of the score. This enabled us to check for floor or ceiling effects, where a large proportion of respondents gave the same answer. We estimated the product moment correlation coefficient between each item and the total score. We also estimated an item by item correlation matrix.

### Dimensionality, factor structure, and internal consistency

These were investigated using the baseline data, collected prior to application of trial treatments. We carried out principal components analysis and calculated eigenvalues, using pairwise deletion for the correlation matrix to handle missing data. We assessed dimensionality visually using a scree plot. After the number of possible factors had been decided, we used a varimax rotation to examine the factor structure and present the factor loadings. Internal consistency was estimated by Cronbach’s alpha coefficient. For VEINES-QOL, we estimated alpha with question 4a for those who worked and without Q4a for all participants.

### Scoring systems

The composite VEINES-QOL score and the symptom subscale were calculated in three ways:

1. using the original, relative method.
2. using the baseline score in this study as the external standard, thus giving, for the baseline data, identical scores for the original relative method and for the external standard.
3. using intrinsic scaling, multiplying by 100 and rounding to the nearest integer to give a more manageable score.

In each case, the total score was set to missing if more than half the questions were not completed and the symptom score was set to missing if more than five questions from the nine parts of questions 1 and question 7 were missing, otherwise the available average of the available questions was taken. The artificial “missing” data for question 4a when participants did not work was included in the count for the full score.

For the original, external, and intrinsic scoring systems, we estimated the mean and standard deviation at baseline, 2 weeks, and 4 months and presented histograms of the distributions. We compared scores at baseline and 4 months using the paired t method and calculated correlations between the scores at each time.

### Construct validity

We used the baseline data for construct validity. To address convergent validity of VEINES-QOL and VEINES-Sym, we estimated their relationship with other quality of life and well-being scales. We estimated correlation coefficients with the SF12 physical and mental subscales and with pain over the past 24 h measured on a 21-point numerical scale. Kendall’s tau b was estimated with self-reported problems with mobility, self-care, interference with usual activities, anxiety/depression, and pain/discomfort, all on three point scales, and pain on a five-point nominal scale. We also estimated the correlation with size and duration of ulcer, using a log transformation as each had a highly skewed distribution.

For divergent validity, we estimated the relationship with two variables unrelated to the ulcer, calculating the correlation with age and the mean score difference between sexes, compared by the two-sample t method.

### Repeatability

This was estimated using the baseline and 2-week data, omitting any participants whose ulcers had healed by 2 weeks. For each individual item, weighted kappa statistics was estimated using quadratic weights. Repeatability of the score was measured by the intra-class correlation coefficient using the test and retest scores. The standard deviation of differences and the coefficient of repeatability (2 × SD of differences) were estimated.

### Responsiveness

Estimation of responsiveness was done using the baseline and 4 month data. We estimated the difference in mean change in score between participants whose ulcers had healed at 4 months and those who remained unhealed, presented as an effect size measured in baseline standard deviation units. Standard errors and confidence intervals were calculated using the two sample t method.

We also measured responsiveness using a responsiveness coefficient. This uses the variance of differences between observations on the same person when there should be no change, between baseline and 2 weeks, and when change should have taken place in at least some participants, between baseline and 4 months. The responsiveness coefficient is then variance for change minus variance for no change divided by variance for change, giving a coefficient between zero and 1.00.

### Software

Principal components analysis was carried out using SPSS version 19 (IBM). All other analyses were carried out using Stata version 10 (Stata Corp., College Station, Texas).

### Ethical approval

This trial was reviewed and approved by the Northern and Yorkshire Research Ethics Committee (09/H0903/25) and all participants gave informed consent. Because of the low-risk nature of this trial (both treatments being assessed were already being used routinely in clinical practice), we did not judge it necessary to have a separate Data Monitoring and Ethics Committee to oversee the trial. Instead, unmasked adverse events data, details of patients no longer receiving randomised treatments, and details of post-randomised exclusions were presented by the trial coordinator (RLA) and trial statisticians (RG, JMB) to independent members of the Trial Steering Committee (chair [Ian Chetter], independent clinician [Brenda King], and independent statistician [Jenny Freeman]) before Trial Steering Committee meetings. This decision was ratified by the study sponsors (National Institute for Health Research Health Technology Assessment programme) and minutes of these meetings were sent to the sponsors. We obtained research governance approval for all centres.

## Results

At baseline, 451 (99.3 %) questionnaires were returned, one was blank and 374 (82.4 %) had complete VEINES-QOL, all others had some missing items. At 2 weeks, 382 questionnaires were returned, four were blank and 289 (63.7 %) had complete VEINES-QOL. At 4 months, 341 questionnaires were returned, one was blank and 242 (53.3 %) had complete VEINES-QOL.

### Individual questions

**Fig. 1:**
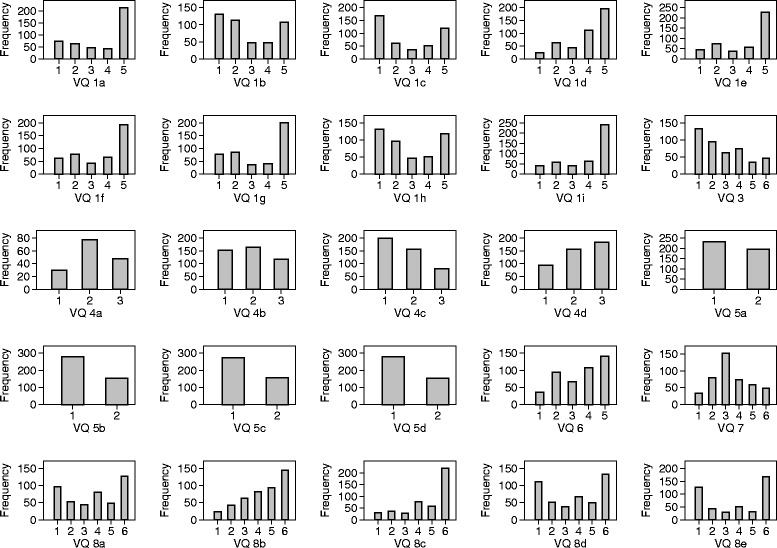
Distribution of each VEINES-QOL question at baseline

The distribution of each scale item at baseline is shown in Fig. 1. The maximum proportion ticking the same box was 55 %, apart from questions with only two options where it was 65 %, so there is nothing to suggest that there were serious floor or ceiling effects. Means and standard deviations for each item are shown in Table 2.

**Table 2:** Individual item mean, standard deviation, and correlation with total score^a^

Question	Mean	SD	Correlation with total VEINES-QOL SCORE, original scoring	Correlation with total VEINES-QOL SCORE, intrinsic scoring
1a	3.59	1.58	0.57	0.56
1b	2.75	1.56	0.62	0.61
1c	2.75	1.69	0.34	0.34
1d	3.88	1.28	0.47	0.45
1e	3.78	1.47	0.50	0.49
1f	3.56	1.53	0.54	0.53
1g	3.46	1.61	0.60	0.59
1h	2.84	1.60	0.41	0.41
1i	3.92	1.41	0.50	0.49
3	2.83	1.67	0.14	0.13
4a	2.12	0.70	0.24	0.25
4b	1.92	0.78	0.66	0.67
4c	1.73	0.75	0.67	0.68
4d	2.21	0.77	0.63	0.63
5a	1.46	0.50	0.65	0.68
5b	1.35	0.48	0.69	0.72
5c	1.37	0.48	0.69	0.72
5d	1.36	0.48	0.69	0.71
6	3.50	1.34	0.75	0.75
7	3.42	1.40	0.65	0.63
8a	3.70	1.91	0.54	0.53
8b	4.36	1.53	0.71	0.70
8c	4.67	1.63	0.63	0.63
8d	3.65	1.98	0.49	0.49
8e	3.71	2.12	0.44	0.44

Note: For a sample of 374 observations, *r* is significant, *P* < 0.05, if *r* > 0.10

^a^For validity, all items should be correlated with the overall score

The 300 inter-item correlations had mean = 0.29, SD = 0.16, range −0.05–0.79. The product moment correlation between each item and the total score is shown in Table 2. All but one of the correlations exceeded 0.20 and the mean correlation was 0.55.

### Internal consistency

For the whole VEINES-QOL scale, Cronbach’s alpha = 0.88 (both with and without question 4a). For the VEINES-Sym symptom scale, alpha = 0.81.

**Fig. 2:**
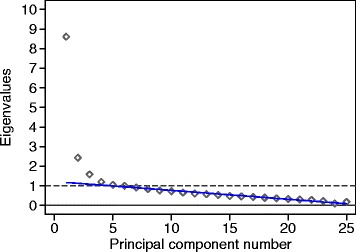
Scree plot for a principal component analysis using the baseline data, showing a line through the points on the right

For principal components analysis, a scree plot of eigenvalue against component number is shown in Fig. 2. It appears that a three-dimensional representation of the data might be informative. The factor loadings with three factors after varimax rotation are shown in Table 3. Factor 1 loads on questions 4, 5, and 6. These are all questions about interference with usual activities. Factor 2 loads on questions 1 and 7 and corresponds exactly to the VEINES-Sym symptom subscale. Even though questions 1h and 1i have loadings less than 0.5, they still have higher loadings for this factor than for the others. Factor 3 loads on question 8, which is about feelings. Question 3, comparing the leg problem now with a year ago, does not load on any factor. We could use this questionnaire to define three subscales, including the symptoms subscale already defined, interference with activities, and feelings produce by the ulcer.

**Table 3:** Factor loadings^a^ for a three-factor model (high loadings in bold)

Question	Factor 1	Factor 2	Factor 3
1a	0.25	0.60	0.10
1b	0.28	0.69	0.07
1c	0.03	0.42	0.06
1d	0.03	0.60	0.15
1e	0.11	0.60	0.15
1f	0.17	0.61	0.15
1g	0.17	0.68	0.16
1h	0.06	0.37	0.31
1i	0.15	0.52	0.19
3	0.02	0.04	0.12
4a	0.77	0.20	−0.01
4b	0.80	0.22	0.02
4c	0.74	0.24	0.12
4d	0.59	0.35	0.07
5a	0.77	0.10	0.15
5b	0.81	0.07	0.23
5c	0.82	0.09	0.19
5d	0.81	0.12	0.16
6	0.64	0.32	0.33
7	0.37	0.56	0.18
8a	0.11	0.21	0.77
8b	0.38	0.39	0.55
8c	0.43	0.17	0.57
8d	0.16	0.16	0.65
8e	0.05	0.09	0.77

^a^High factor loadings, e.g. >0.5, indicate that the question is an important component of the factor

### Composite scale

Table 2 shows the correlations of individual items with the intrinsic-scored VEINES-QOL. The mean correlation is 0.55 for the original VEINES-QOL and 0.55 for the intrinsic scoring (*P* = 0.9, paired *t* test).

The results at baseline, after 2 weeks, and after 4 months are shown in Table 4. As the internal and external standards are the same for the original coding and the external standard at baseline, they give identical scores. After baseline, they are no longer identical. The increase in the mean external standard score over 4 months from 50.0 to 54.8 is highly significant, *P* < 0.001, showing that an improvement in quality of life has taken place. This is also true of the intrinsic coding.

**Table 4:** Composite scores^a^ using three different scaling methods

	Baseline		Two weeks		Four months	
Scale	Mean	SD	Mean	SD	Mean	SD
VEINES-QOL						
Original	50.0	10.0	50.0	10.0	50.0	10.0
External standard^b^	50.0	10.0	49.2	10.2	54.5	11.1
Intrinsic	53.6	22.1	51.8	22.7	62.9	24.8
Symptoms						
Original	50.0	10.0	50.0	10.0	50.0	10.0
External standard^b^	50.0	10.0	50.3	10.3	54.8	10.6
Intrinsic	58.4	23.0	59.0	23.8	69.2	24.3

^a^different methods of forming the score may produce different numerical values

^b^At baseline the external standard score is identicalto the baselinescore, because the baseline dataset forms the standard reference set

**Fig. 3:**
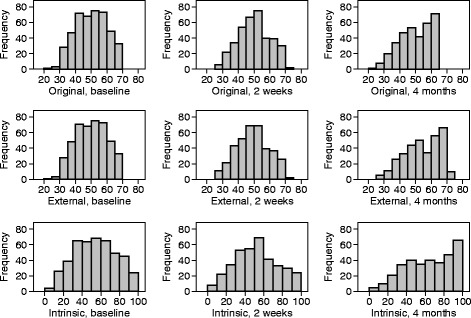
Distribution of total score at three times using different scoring methods

**Fig. 4:**
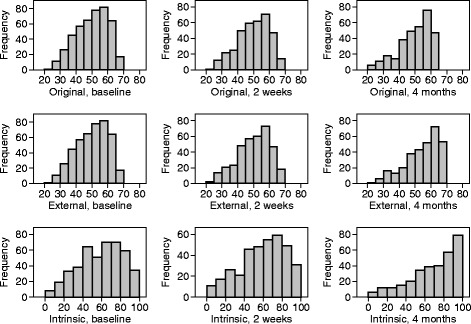
Distribution of symptom score at three times using different scoring methods

The distribution of the total score at each time is shown in Fig. 3. At baseline and at 2 weeks, the distribution is symmetrical. However, at 4 months the distribution has pronounced negative skewness. This might be expected in a disease-specific measure for a disease from which total recovery, at least in terms of healing of the ulcer, is frequent. By 4 months, more than half the reference ulcers had healed. The distribution of the symptom score at each time is shown in Fig. 4. The distribution is negatively skewed at each time and very much so at 4 months.

**Fig. 5:**
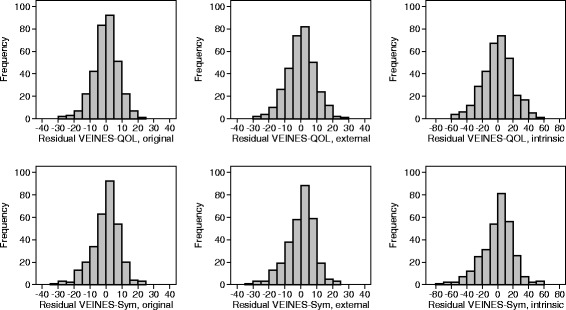
Distribution of residual VEINES-QOL and symptom score at 4 months after regression on score at baseline using different scoring methods

Figure 5 shows the distribution of the residuals of the scores at 4 months after regression on score at baseline. These have more symmetrical distributions than those in Fig. 4 and show that the scales are suitable for use in statistical analysis without transformation, whichever scoring system is used.

Correlation coefficients between each pair of the three scoring methods were 1.00 at each time. Any relationship with one score will apply to the others, too.

### Construct validity

First we looked at convergent validity: does the score have positive relationships with other quality of life and well-being scales? We compared the VEINES-QOL to the SF-12 quality of life measure. At baseline, the mean (SD) were 38.4 (11.2) for the physical component summary score and 49.6 (11.3) for the mental component summary score. Our sample has considerably poorer quality of physical life than the SF-12 reference sample and has very similar quality of mental life. Correlation coefficients between the SF-12 components and all scoring systems are shown in Table 5. This shows that the correlation is stronger with the total VEINES-QOL than with VEINES-Sym, and that all correlations are highly significant.

**Table 5:** Correlations^a^ of VEINES-QOL and VEINES-Sym at baseline with SF12 components, reported pain and other problems, size and duration of the ulcer, and age

	VEINES-QOL		VEINES-Sym	
	Original or external	Intrinsic	Original or external	Intrinsic
SF12 Physical	*r* = 0.58	*r* = 0.58	*r* = 0.43	*r* = 0.42
SF12 Mental	*r* = 0.58	*r* = 0.57	*r* = 0.44	*r* = 0.44
Pain (0–100)	*r* = −0.60	*r* = −0.58	*r* = −0.60	*r* = −0.59
Pain (five categories) (Kendall’s tau b)	*τ*_*b*_ = −0.42	*τ*_*b*_ = −0.41	*τ*_*b*_ = −0.43	*τ*_*b*_ = −0.42
Pain or discomfort (three categories)	*τ*_*b*_ = −0.41	*τ*_*b*_ = −0.40	*τ*_*b*_ = −0.40	*τ*_*b*_ = −0.39
Mobility (three categories)	*τ*_*b*_ = −0.39	*τ*_*b*_ = −0.39	*τ*_*b*_ = −0.31	*τ*_*b*_ = −0.29
Problems with self-care (three categories)	*τ*_*b*_ = −030.	*τ*_*b*_ = −0.30	*τ*_*b*_ = −0.23	*τ*_*b*_ = −0.23
Interference with usual activities (three categories)	*τ*_*b*_ = −0.41	*τ*_*b*_ = −0.42	*τ*_*b*_ = −0.25	*τ*_*b*_ = −0.25
Anxiety or depression (three categories)	*τ*_*b*_ = −0.36	*τ*_*b*_ = −0.36	*τ*_*b*_ = −0.30	*τ*_*b*_ = −0.28
Log duration of ulcer at baseline	*r* = −0.02 (*P* = 0.6)	*r* = −0.03 (*P* = 0.6)	*r* = −0.02 (*P* = 0.6)	*r* = −0.02 (*P* = 0.7)
Log area of ulcer at baseline	*r*=−0.10 (*P* = 0.03)	*r* = −0.10 (*P* = 0.04)	*r* = −0.09 (*P* = 0.07)	*r* = −0.09 (*P* = 0.07)
Age	*r* = 0.25	*r* = 0.24	*r* = 0.26	*r* = 0.26

All *P* < 0.0001 except duration and area of ulcer

*r* = product moment correlation coefficient, *τ*_*b*_ = Kendall’s tau b coefficient of rank correlation

^a^correlation of score with generic quality of life scales, pain, and difficulties indicate convergent validity, lack of correlation or low correlation with variables which should not be related to quality of life, such as age and duration of ulcer, would indicate divergent validity

Pain over the past 24 h was recorded on a 21-point pain scale where the result is a score from 0 to 100, and also on a five-point nominal pain scale with categories “No pain”, “Very mild pain”, “Mild pain”, “Severe pain”, “Very severe pain”. Higher pain was related to poorer quality of life (Table 5). We also asked a three category question about general pain or discomfort: “I have no pain or discomfort”, “I have moderate pain or discomfort”, “I have extreme pain or discomfort”. This had rank correlations with VEINES-QOL of tau b = −0.41 and with VEINES-Sym tau b = −0.40, both *P* < 0.0001.

We also asked whether quality of life measured by VEINES-QOL/Sym is related to other specific aspects of overall quality of life: problems with mobility, problems with self-care, interference with usual activities, and anxiety/depression. All showed significant negative rank correlations of moderate strength with both VEINES-QOL and VEINES-Sym (Table 5).

We also considered size and duration of the ulcer. These both had highly skewed distributions so were log transformed before calculation of correlation coefficients. Long duration and large ulcers might reduce the quality of life. Correlations were negative, as expected, but close to zero (Table 5).

To address divergent validity, we examined the relationship between VEINES-QOL/Sym with variables unrelated to the ulcer: age and sex. The correlations with age are weak though highly significant (Table 5). Mean scores were very similar for men and women and there was no evidence of any systematic difference. For VEINES-QOL, the mean scores were 49.6 (women) and 50.4 (men) (*P* = 0.4) using original or external scoring, 52.6 (women) and 54.5 (men) (*P* = 0.4) using intrinsic scoring. For VEINES-Sym, the mean scores were 50.0 for both men and women (*P* = 1.0) using original or external scoring and 58.5 (women) and 58.3 (men) (*P* = 0.9) using intrinsic scoring.

### Repeatability

We addressed repeatability using the baseline and 2-week data. No participants were reported to have healed ulcers in this time, so all were used to estimate agreement. For each individual questionnaire item, weighted kappa statistics using quadratic weights are shown in Table 6. Kappas are between 0.42 and 0.73, so all indicate moderate or good agreement and none are unacceptable. The highest kappa is for Question 7, pain over the past 24 h.

**Table 6:** Kappa statistics for 2-week test-retest reliability for each question in VEINES-QOL^a^

Question	Kappa	Standard error	Significance
1a	0.56	0.054	*P* < 0.0001
1b	0.58	0.053	*P* < 0.0001
1c	0.44	0.053	*P* < 0.0001
1d	0.67	0.054	*P* < 0.0001
1e	0.54	0.053	*P* < 0.0001
1f	0.68	0.054	*P* < 0.0001
1g	0.59	0.054	*P* < 0.0001
1h	0.59	0.053	*P* < 0.0001
1i	0.62	0.054	*P* < 0.0001
3	0.56	0.052	*P* < 0.0001
4a	0.58	0.106	*P* < 0.0001
4b	0.59	0.053	*P* < 0.0001
4c	0.53	0.053	*P* < 0.0001
4d	0.46	0.053	*P* < 0.0001
5a	0.42	0.055	*P* < 0.0001
5b	0.46	0.055	*P* < 0.0001
5c	0.55	0.054	*P* < 0.0001
5d	0.48	0.054	*P* < 0.0001
6	0.63	0.051	*P* < 0.0001
7	0.73	0.052	*P* < 0.0001
8a	0.55	0.052	*P* < 0.0001
8b	0.64	0.052	*P* < 0.0001
8c	0.67	0.052	*P* < 0.0001
8d	0.61	0.052	*P* < 0.0001
8e	0.71	0.052	*P* < 0.0001

^a^Individual questions should be repeatable, i.e. answers on different occasions should be related if the questionnaire is valid

For the composite scale and subscale, intra-class correlation coefficients are shown in Table 7. For the full VEINES-QOL this was ICC = 0.80 and for VEINES-Sym ICC = 0.75. Table 7 also shows the within-subject standard deviation and the coefficient of repeatability, the value within which 95 % of differences between pairs of measurements on the same person are expected to be found. It is noticeable that the ICCs are slightly larger for the original, internally standardised scoring than for the external or intrinsic standardised scores. This is because the internally standardised scores cannot change overall between observations.

**Table 7:** Intraclass correlation coefficients, within subject standard deviation, and repeatability coefficient for 2-week test-retest reliability for VEINES-QOL and VEINES-Sym

Scale and scoring	ICC^a^	SE	95 % CI	Within-subject SD	Repeatability coefficient^b^
VEINES-QOL					
Original	0.80	0.019	0.76–0.83	4.5	12.5
External standard	0.79	0.019	0.75–0.83	4.6	12.8
Intrinsic	0.78	0.020	0.74–0.82	10.6	29.4
VEINES-Sym					
Original	0.75	0.022	0.71–0.79	5.0	13.8
External standard	0.75	0.022	0.71–0.79	5.1	14.1
Intrinsic	0.74	0.023	0.69–0.79	11.9	33.0

^a^ICC = intraclass correlation coefficient, which lies between 0 and 1.0. For acceptable repeatability this should be greater than 0.070

^b^95 % of pairs of replicate measurements on the same person will be less than this, so a larger difference would indicate a change in quality of life

### Responsiveness

At 4 months, we had VEINES-QOL questionnaires from 198 participants who had healed by 4 months and 120 questionnaires from participants who had not healed (187 and 111 for VEINES-Sym). Table 8 shows the mean increases in VEINES-QOL and VEINES-Sym. For both scales and for external and intrinsic coding, the mean increase was positive in both groups and greater for the healed group. In each case this difference was significant. However, there was considerable variability and the differences between healed and unhealed were small. For external coding, in terms of the standard deviation of the baseline score (10.0), this represents 0.26 standard deviations for VEINES-QOL and 0.23 for VEINES-Sym. For intrinsic coding, this represents 0.25 and 0.23 respectively. Responsiveness coefficients showed moderate responsiveness. This coefficient measures how much change takes place over the period in the underlying quantity being measured, not whether it is related to changes in the ulcer.

**Table 8:** Increase in VEINES-QOL scores for those who have healed and those who have not healed after 4 months, with responsiveness coefficient

Scale	Healed		Not healed		Healed minus not healed^a^			Responsiveness^b^
	Mean	SD	Mean	SD	Difference	95 % CI	P	
VEINES-QOL, external	4.7	9.7	2.1	7.9	2.6	0.5–4.7	0.01	0.50
VEINES-Sym, external	4.8	9.7	2.5	9.1	2.3	0.1–4.6	0.04	0.44
VEINES-QOL, intrinsic	9.8	22.1	4.3	17.2	5.5	0.8–10.2	0.02	0.48
VEINES-Sym, intrinsic	10.6	22.7	5.4	20.8	5.2	0.0–10.5	0.05	0.42

^a^Bigger changes in those who have healed than in those who have not healed indicate that healing is associated with change in reported quality of life

^b^a value greater than zero (maximum is 1.00) indicates that the scale responds to changes over time

## Discussion

This is the first validation study of VEINES-QOL/Sym in venous leg ulcers. It found the instrument to be repeatable and to have construct validity. We found that the VEINES-Sym subscale was exactly reproduced by factor analysis. We found that, using either external or intrinsic scaling, VEINES-QOL/Sym was responsive to ulcer healing.

The original validation study [12] was in a mixed group of patients with venous disease and the second was in deep vein thrombosis [13]. Since VenUS IV began, three other validity studies of VEINES-QOL/Sym have been published:

- a Norwegian version in deep vein thrombosis [19], with 74 participants with repeat questionnaires after 7–10 days from obtained from 40 of them.
- a Turkish version in chronic venous insufficiency [20], using 118 patients from the cardiovascular surgery units of three hospitals.
- another, apparently independent, Turkish version in chronic venous insufficiency [21], where of 100 patients included, 30 were given the questionnaire twice with 24-hour intervals for test-retest.

### Individual questions

These studies were all in agreement that that the scale was reliable, reporting alpha between 0.86 and 0.94 for VEINES-QOL and between 0.81 and 0.88 for VEINES-Sym. One study reported means and standard deviations for individual questionnaire items [19]. One study reported principal components analysis or factor analysis [20]. This reported a solution with seven components, which explained 67.5 % of the total variation cumulatively. They do not say how the number of components was decided, but if they used the Kaiser criterion of eigenvalue >1.0, this would be very similar to our six components for which eigenvalues >1.0 (Fig. 2), which together accounted for 63.4 % of the total variance.

In this study all but one item-scale correlation exceeded 0.20 and the mean correlation was 0.55, in line with other studies which reported that all items had correlations with VEINES-QOL which were greater than 0.20 [12, 13], between 0.29 and 0.78 [19], and between 0.27 and 0.62 [20].

Only one study [20] reported on the distribution of VEINES-QOL/Sym scores, giving mean, SD, median, skewness, kurtosis, and range. These indicated fairly symmetrical distributions. For each scale they reported mean = 50 and standard deviation = 10, which was bound to happen by definition.

We reported that individual items all showed moderate or good kappa-repeatability. No other study quoted kappa statistics.

For the composite scale and subscale, intra-class correlation coefficients, 0.80 and 0.75, were smaller than the ICCs in the original validation study [12], 0.91 and 0.87 respectively for their English-speaking sample. Correlation depends on the variation between subjects. We cannot compare this between the two samples due to the internal referencing, but it is likely that our single-diagnosis venous leg ulcer sample is less variable than Lamping’s multi-diagnosis sample. Other studies reported 0.87 and 0.87 [13], 0.88 and 0.83 [19], and 0.97 and 0.93 [21], which may be the result of using only 24 h as the interval between questionnaires.

### Construct validity

We reported correlations with SF-12 of 0.58 for the physical component and 0.43 for the mental component. Using SF-36, the original study [12] reported correlations with the physical component of 0.62 and 0.46 with VEINES-QOL and VEINES-Sym respectively and with the mental component 0.19 and 0.15. In deep vein thrombosis, the corresponding correlations were 0.63, 0.49, 0.37, and 0.29 [13] and in venous insufficiency they were 0.7, 0.66, 0.60, and 0.50 [21]. Also in venous insufficiency, there were significant correlations with all eight subscales of SF-36, stronger for VEINES-QOL than for VEINES-Sym in every case [20]. There were also significant relationships with EQ-5D [19]. There were the expected relationships with several clinical scores and classifications.

No one reported differences between sexes. We found a weak positive correlation with age in contrast to reported correlations of similar magnitude but opposite direction [12], much smaller positive correlations [13], and no evidence of a relationship [20].

### Responsiveness

Only two studies [12, 13] reported evidence of responsiveness, but as their samples represent very different spectra of disease and indicators of change, it is impossible to compare our results directly. None of the other studies reported on responsiveness.

Although we have evidence that VEINES-QOL scores increased over time (Table 7), there was still considerable variability, even after healing. As Fig. 4 shows, even when the majority of participants had healed, there was great variation in scores. The ulcer may have healed, but the underlying disease remains and participants are at high risk of a recurrence of ulceration. We should not think that when the ulcer has gone our work is done.

### Quality of life in venous leg ulcer patients

The poor quality of life of these venous leg ulcer patients is shown by their mean SF-12 physical component score of 38.4. For comparison, a review of SF-12 and SF-36 scores in cardiovascular disease [22] reported the mean physical component to be 44.4 in hypertension patients, 38.9 in ischaemic heart disease, and 35.9 in heart failure, so these patients had similar physical quality of life to patients with ischaemic heart disease. For mental quality of life, our participants’ mean score was 49.6, very similar to 50 in the standard population. This suggests that, although some reported depression or anxiety, this was at a similar level to the general population.

### Scaling method

We have shown that the original scaling method has a disadvantage, in that it always produces the same mean and standard deviation and so cannot be used to compare different studies and would be difficult to use to investigate changes over time. We think that either the use of an external standard or our intrinsic scoring system would be better. The external standard has the advantage that we can obtain a T-score, where the mean and standard deviation in the reference sample are 50 and10 and all other samples can be compared to that. For VEINES-QOL/Sym, however, we could not do this using a normal reference group, as the questions are relevant only to people with a leg problem. The only reference group we have is our own leg ulcer group and this may not be relevant for patient groups with other venous conditions. For anybody wishing to do this, we have published our means and standard deviations at baseline. The intrinsic scoring method could be used for any venous disease group and used to compare groups and to study changes over time and this is what we would prefer for future use. It appears to have good statistical properties for a research tool. Its distribution at 4 months follow-up appears to be the closest to Normal of the three scoring systems, for example.

## Conclusions

The VEINES-QOL/Sym instrument is valid, reliable, and responsive for people with venous leg ulcers. Scaling with the external standard (even though it is internal for the baseline VEINES-QOL) performed very slightly better than the intrinsic standard. However, there is no way to tell whether this would be true for other samples, especially those with a different disease mix. The intrinsic scoring method should work in the same way for other samples and so we propose that this approach should be used in future. This would enable scores to be compared easily between studies. We recommend that this approach should be used in future venous ulcer studies. The adoption of the intrinsic scoring system would also enable VEINES-QOL/Sym to be used for monitoring quality of life in individuals with venous leg ulcers.
